# Returning to the Basics: Embracing Oral Rehydration Therapy During an Intravenous Fluid Shortage After Hurricane Helene

**DOI:** 10.7759/cureus.80146

**Published:** 2025-03-06

**Authors:** Christopher Mikulas, Krina Patel, Oshin Rai, Elias Couto Barbosa, Ronald L Mars

**Affiliations:** 1 Internal Medicine, University of Florida College of Medicine – Jacksonville, Jacksonville, USA; 2 Osteopathic Medicine, Nova Southeastern University Dr. Kiran C. Patel College of Osteopathic Medicine, Davie, USA; 3 Nephrology, University of Florida College of Medicine – Jacksonville, Jacksonville, USA

**Keywords:** electrolyte replacement, fluid resuscitation, intravenous fluid therapy, oral rehydration solution, oral rehydration therapy (ort)

## Abstract

In developed countries, the use of intravenous (IV) fluid for fluid resuscitation and other routine medical or surgical indications has become standard practice, with its availability often taken for granted. However, natural disasters can disrupt supply chains, leading to shortages. In September 2024, Hurricane Helene rendered the Baxter Healthcare facility in North Carolina inoperable, halting production and disrupting the supply of IV fluid in the U.S. In contrast, countries with limited access to IV fluid have long relied on oral rehydration therapy (ORT) as the cornerstone of fluid resuscitation and replacement. The well-documented efficacy of ORT provided a viable alternative to IV fluid for managing volume and electrolyte loss in the aftermath of the hurricane. This case series presents two patients, one with nephrogenic diabetes insipidus and the other with acute tubular necrosis due to dehydration, who were successfully treated with ORT, reinforcing its potential role as a practical solution during IV fluid shortages.

## Introduction

In September 2024, Hurricane Helene caused widespread devastation and the southeastern U.S. Baxter Healthcare, one of the world's leading suppliers of intravenous (IV) fluid, sustained catastrophic damage to its North Carolina (NC) facility [1]. This disaster resulted in a significant shortage of IV fluid and dialysate for peritoneal dialysis, critically impacting hospitals nationwide. In response to the anticipated supply deficit, the University of Florida (UF) Health pharmacists issued an urgent directive to all providers, requesting the restriction of IV fluid use to emergency cases only, such as hemodynamically unstable patients and patients who cannot tolerate oral rehydration therapy (ORT). Consequently, clinicians, particularly those in the Nephrology service, adapted by utilizing ORT to manage fluid, electrolyte, and insensible losses in nonemergent scenarios. In this case series, we discuss two patients who required ORT to address significant metabolic abnormalities amid this severe IV fluid shortage.

## Case presentation

Case 1

A was a 49-year-old, 60 kg female patient with a past medical history of type 2 diabetes mellitus and stage 3a chronic kidney disease who presented to the emergency department (ED) for altered mental status and was admitted to the medical intensive care unit (MICU) for mixed hyperosmolar, hyperglycemic diabetic ketoacidosis in the setting of left lung abscess and Enterococcus faecalis bacteremia for which she completed a course of antibiotics. Unfortunately, the patient’s mentation did not improve despite aggressive care in the MICU. It was suspected this altered mentation was due to diffuse hypoxic ischemic injury seen on a computed tomography (CT) scan of the head. A percutaneous endoscopic gastrostomy (PEG) tube was placed for fluid and nutritional support. Her hospital course was complicated by large-volume diarrhea due to *Clostridioides difficile* infection, which was treated with a course of oral vancomycin. The patient was transferred to the hospitalist service in stable condition for further management.

Nephrology was consulted for azotemia and persistent metabolic alkalosis. On physical examination, the patient was cachectic with dry mucous membranes and flat neck veins. Vital signs were significant for a blood pressure of 111-135/65-80 mmHg over the past 24 hours. A bedside ultrasound revealed an inferior vena cava diameter of less than 1.5 cm and greater than 50% collapse with inspiration, suggesting intravascular volume depletion. Laboratory results were significant for serum sodium (Na) of 134 mEq/L, blood urea nitrogen (BUN) of 113 mg/dL, creatinine (Cr) of 1.14 mg/dL, potassium (K) of 3.1 mEq/L, sodium bicarbonate (NaHCO3) of 38 mEq/L, and a pH of 7.52.

Urine chemistries revealed a Na of 54 mEq/L, Cr of 11.2 mg/dL, and BUN of 532 mg/dL with a protein-to-creatinine ratio of 2.59 mg/mg. Renal failure indices, including urine to serum Cr ratio of 9.82, renal failure index of 5.5, fractional excretion of sodium of 4.1%, and fractional excretion of urea of 47.9%, were consistent with intrinsic renal disease. It was suspected that the patient was in a volume-contracted state that had led to acute tubular necrosis.

She averaged 1.8 L of urine output (UOP) and an estimated 600 mL of insensible losses per day. To expand the patient’s intravascular volume while replacing urinary electrolyte losses, one packet of chicken broth was dissolved in 800 mL of water and administered through the PEG tube every eight hours. Twenty-four hours later, the patient’s BUN had decreased to 83 mg/dL, NaHCO3 to 28 mEq/L, Cr to 0.75 mg/dL, and K to 3.1 mEq/L. Her potassium levels were replenished using a potassium chloride solution. Urine chemistries were subsequently repeated to assess intravascular volume status and electrolyte losses, and further managed with a chicken broth solution.

Case 2

B was a 61-year-old, 100 kg male patient with a past medical history of hypertension, heart failure with preserved ejection fraction, and stage 3a chronic kidney disease who presented to the ED for altered mental status. Vital signs on presentation were significant for fever and tachycardia. On physical examination, the patient was somnolent and disoriented without focal neurologic deficits. The initial workup revealed leukocytosis and acute kidney injury with a Cr of 2.94 mg/dL, BUN of 31 mg/dL, and creatine kinase (CK) of 1,140 U/L. Dipstick urinalysis demonstrated moderate blood, large leukocyte esterase, 100 mg/dL of protein, two isomorphic red blood cells per high power field, and 4+ bacteria with urine culture growing mixed flora. Imaging included an unremarkable chest X-ray and CT of the head. Given a concern for meningitis and encephalitis, a lumbar puncture was attempted but was unsuccessful. Empiric IV cefepime, vancomycin, and acyclovir were given. Meningitis and encephalitis were ruled out after a second attempt at lumbar puncture was successful and cerebrospinal fluid studies were unremarkable. Empiric antibiotics and acyclovir were then discontinued. The patient was admitted to the internal medicine service for further management.

He developed hypernatremia to 149 mEq/L on day two of hospitalization with an increase to 156 mEq/L on day three despite enteral nutrition and free water flushes via a Dobhoff tube (Avanos Medical, Inc., Georgia, US). The Nephrology service was consulted for refractory hypernatremia. Urine chemistries were significant for Na of 61 mEq/L, K of 23 mEq/L, BUN of 478 mg/dL, and Cr of 83 mg/dL. Given the concern for central diabetes insipidus, desmopressin was administered with no change in serum Na, urine Na, or UOP. Thus, a diagnosis of nephrogenic diabetes insipidus was made.

With a goal serum Na reduction of 6 mEq/L per day, a free water deficit of 2.4 L was calculated and replaced with 300 mL free water flushes every 3 hours. The patient’s urinary electrolyte losses also needed to be replaced. Based on a daily UOP of 2.4 L and an estimated 1 L of insensible losses, the total daily Na and K loss was calculated to be 241.6 mEq. To address this, replacement therapy was planned using eight standard 20 oz bottles of Gatorlyte (PepsiCo, Inc., New York, US) per day, administered as one bottle every three hours. After three days of serial monitoring of urine and serum chemistries, the correction of the calculated free water deficit with free water flushes, and electrolyte replenishment using Gatorlyte, the patient’s serum Na level normalized and Cr returned to baseline.

Details of management

Electrolyte losses were measured in spot urines with total losses extrapolated to 24-hour UOP. The volume and electrolyte content of insensible losses, diarrhea, or other body fluids were estimated at 10 mL/kg/day with 35 mEq of Na and 5 mEq of K per L [10,11]. Contents of Gatorlyte included 490 mg of Na and 350 mg of K.

Millequivalents of Na and K were calculated using the formula: $\text{mEq} = \frac{\text{mg} \times \text{valence (or 1)}}{\text{atomic weight}}$.

Therefore, each standard bottle of Gatorlyte contained 21.3 mEq of Na and 9 mEq of K. Similarly, each packet of chicken bouillon provided 1,100 mg of Na (or 48 mEq).

Patient A developed azotemia, metabolic alkalosis, and acute tubular necrosis due to a prolonged volume-contracted state. Thus, fluid resuscitation was imperative. This was achieved with chicken bouillon packets. Per urine chemistries, patient A lost 54 mEq/L of Na in her urine with 1.8L UOP daily, equating to 97.2 mEq of Na per day. The calculations were as follows:

\[ \text{Urine Na loss} = \text{UOP} \times \text{urine Na} \] \[ = 1.8 \,\text{L/day} \times 54 \,\text{mEq/L} \] \[ = 97.2 \,\text{mEq/day} \]

Patient A also had 600 mL of insensible losses per day with approximately 35 mEq of Na/L of insensible losses or 21 mEq per day.

\[ \text{Insensible Na loss} = \text{insensible losses/day} \times 35 \,\text{mEq/L} \] \[ = \frac{[10 \,\text{mL/weight (kg)}]}{\text{day}} \times 35 \,\text{mEq/L} \] \[ = \frac{[10 \,\text{mL}/(60 \,\text{kg})]}{\text{day}} \times 35 \,\text{mEq/L} \] \[ = \frac{[600 \,\text{mL}]}{\text{day}} \times 35 \,\text{mEq/L} \] \[ = \frac{[0.6 \,\text{L}]}{\text{day}} \times 35 \,\text{mEq/L} \] \[ = 21 \,\text{mEq/day} \]

Thus, the plan was to volume resuscitate patient A with three chicken broth packets in 2400 cc of free water per day. One packet was dissolved in 800 mL of free water and given via the PEG tube every eight hours to achieve this goal.

Patient B’s hypernatremia refractory to free water flushes and desmopressin was concerning for nephrogenic diabetes insipidus. The management was to stop the suspected offending agent (acyclovir) and correct the electrolyte and free water deficits. The urine chemistries revealed a Na of 61 mEq/L and K of 23 mEq/L. Given 2.4 L of UOP and 1 L of insensible losses per day, an estimated 241.6 mEq of Na and K needed to be replaced. This was to be achieved with Gatorlyte with each bottle containing 30 mEq of Na and K. Thus, eight bottles of Gatorlyte per day would replace this deficit. This was accomplished with one bottle every three hours via Dobhoff tube.

The calculations were as follows:

\[ \text{Urine Na and K loss} = \text{UOP} \times [\text{urine Na} + \text{urine K}] \] \[ = 2.4 \,\text{L/day} \times [(61 \,\text{mEq/L}) + (23 \,\text{mEq/L})] \] \[ = 2.4 \,\text{L/day} \times [84 \,\text{mEq/L}] \] \[ = 201.6 \,\text{mEq/day} \]

\[ \text{Insensible Na and K loss} = \text{insensible losses/day} \times (35 \,\text{mEq/L} + 5 \,\text{mEq/L}) \] \[ = \frac{[10 \,\text{mL/weight (kg)}]}{\text{day}} \times 40 \,\text{mEq/L} \] \[ = \frac{[10 \,\text{mL/weight (100 kg)}]}{\text{day}} \times 40 \,\text{mEq/L} \] \[ = \frac{[1000 \,\text{mL}]}{\text{day}} \times 40 \,\text{mEq/L} \] \[ = \frac{[1 \,\text{L}]}{\text{day}} \times 40 \,\text{mEq/L} \] \[ = 40 \,\text{mEq/day} \]

A free water deficit of 2.4 L was calculated using the equation: $\text{Free water deficit} = \text{total body water} \times \left( \frac{\text{serum Na} - \text{desired Na}}{\text{desired Na}} \right)$, where total body water equals body weight in kg multiplied by 0.5 [12]. A modest Na reduction of 6 mEq/L/day was chosen to avoid fluid overload in the setting of the patient's history of heart failure with preserved ejection fraction. This free water deficit was corrected with 300 mL free water flush every three hours.

## Discussion

The modern era of oral rehydration solution (ORS) originated in the 1940s when pediatricians recognized the importance of early oral feedings in managing diarrhea during infancy and childhood [2]. The scientific basis for ORS was established in the 1950s through physiological research that elucidated the mechanism of the sodium-glucose cotransporter 1 (SGLT-1) in the intestinal cells [3]. Subsequent studies conducted in Dhaka and Calcutta demonstrated that the SGLT-1 remained functional in patients with cholera. As a result, ORS effectively hydrated these patients, allowing for the conservation of nearly 80% of IV fluids by utilizing the oral route for hydration. The use of ORS significantly reduced morbidity and mortality from acute diarrhea, particularly after the World Health Organization (WHO) adopted and promoted it [4].

Standard ORS developed by the WHO contained 90 mEq/L of Na with a total osmolarity of 310 mOsm/L [5]. One adverse effect of standard ORS was a high “osmotic load” on the small intestine, which was found to worsen diarrhea [6]. Numerous studies have been implemented to develop an “improved” ORS that is as safe and effective as standard ORS with reduced osmotic stress on the intestine. This was achieved by lowering the solution’s sodium and glucose concentration and total osmolarity [5]. Substituting glucose polymers for simple glucose decreased the solution’s osmolarity while providing favorable glucose-to-sodium ratios [6]. In a combined analysis, stool output with this modified ORS was reduced by about 20% and vomiting by about 30%, while proving to be as safe and effective as standard ORS for use in children and adults with cholera. Due to the enhanced effectiveness of the reduced-osmolarity ORS, the WHO and the United Nations Children's Fund (UNICEF) updated their recommendations to advocate for this modified ORS for diarrhea management across all etiologies and age groups (Table 1) [5].

**Table 1:** Composition of oral rehydration solutions (ORS) and other fluids ORT = Oral rehydration therapy, WHO = World Health Organization, UNICEF = United Nations Children's Fund, Glu = glucose, DW = dextrose, FOS = fructose oligosaccharide, F = fructose, Suc = sucrose. Normal serum plasma osmolarity is 275-295 mOsm/kg [6]. Details of rehydration solutions: Ceralyte (Cera Products, Inc., Georgia, US); EquaLyte (First Wave Products Group, LLC, New York, US); Jianas Brothers ORS (Jianas Brothers Packaging Company, Missouri, US); Liquilyte (PharmaLex, Germany); Pedialyte (Abbott Laboratories, Illinois, US); Nutrisca (Procter & Gamble, Ohio, US); Gatorade (PepsiCo, Inc., New York, US)

ORT		Carbohydrate (g/L)	Sodium (mEq/L)	Potassium (mEq/L)	Base (mEq/L)	Osmolarity (mOsm/L)
WHO/UNICEF ORS formulae	Standard formula	20 (Glu)	90	20	30	310
	Reduced-osmolarity formula	13.5 (Glu)	75	20	30	245
Rehydration solutions	CeraLyte 70	40 (rice starch polymers)	70	20	30	220-235
	CeraLyte 90	40	90	20	30	260
	EquaLyte	30 (DW, FOS)	78.2	22.3	30	290
	Jianas Brothers ORS	20 (Glu)	90	20	10	≥300
	Liquilyte	25 (DW)	45	20	30	250
	Pedialyte	25 (Glu or DW, F)	45	20	30	250
	Rehydralyte (Nutrisca)	25 (DW)	75	20	30	310
Other fluids	Gatorade (powder)	58 (F, Suc, Glu)	20	3	3	330-380
	Chicken broth	1-3	30-39	8-12	1-3	200-350
	Tea	0	0-10	0	0	18
	Water	0	0-10	0	0	0-18

ORT can range from simple home remedies like chicken or beef broth dissolved in water to match fluid and sodium losses to more advanced commercial solutions like Gatorade (Table 1). Most formulae combine iso-osmolar glucose, electrolytes, and base (e.g., citrate) to treat dehydration and metabolic acidosis. Glucose enhances the absorption of sodium, and consequently water, in the small intestine on a 1:1 molar basis. Sodium and potassium are essential for replenishing the body’s losses of these critical ions. Additionally, citrate helps to correct acidosis resulting from diarrhea and dehydration [5].

While ORT is predominantly utilized in nations with limited resources, developed countries have come to rely on IV fluids to manage hypovolemia and electrolyte deficits. However, IV fluid shortages in the U.S. have renewed interest in ORT [7]. In September 2017, Hurricane Maria caused a significant disruption in the production of IV fluid in Puerto Rico, which supplied 44% of the IV fluid used in the U.S. [8]. In September 2024, Hurricane Helene left the Baxter Healthcare facility in NC inoperable, bringing IV fluid production for 60% of the U.S. to an abrupt halt [9]. These disasters resulted in the urgent need for ORT as a replacement for IV fluid.

In these two cases, Patients A and B were already receiving nutritional supplementation with other options like osmolyte, Nepro (Abbott Laboratories, Illinois, US) and Novasource (Nestlé Health Science, Switzerland). Additionally, neither patient had diarrhea nor associated metabolic acidosis when evaluated by the Nephrology service. As such, hemodynamic support and electrolyte correction were prioritized. The decision of which ORT to use was based on availability. Gatorlyte was used for Patient B to replenish Na and K losses. Since it was not available for Patient A, chicken broth was utilized for intravascular volume expansion, and potassium chloride was supplemented as needed.

## Conclusions

In developed countries, the consistent availability of IV fluid is often taken for granted. This assumption was challenged by the devastating impact of Hurricane Helene on the southeastern U.S., which disrupted IV fluid production at the Baxter Healthcare NC facility. Fortunately, the proven efficacy of ORT, particularly in regions with limited resources, has established a therapeutic precedent for using ORT as a viable alternative to IV fluid. These two case reports aim to reinforce that precedent and provide a valuable reference for clinicians encountering similar challenges in their practice.
